# Extranodal NK/T-Cell Lymphoma, Nasal Type, Presenting as an Isolated Oral Manifestation

**DOI:** 10.3390/dj14020129

**Published:** 2026-02-23

**Authors:** Andrea Kanizsai, Ágnes Bán, László Kereskai, Árpád Szomor

**Affiliations:** 1 Maxillofacial Division, Oral and Maxillofacial Surgery, Department of Dentistry, Medical School, University of Pécs, Rákóczi Street 2, H-7623 Pécs, Hungary; 2 Division of Periodontology, Oral and Maxillofacial Surgery, Department of Dentistry, Medical School, University of Pécs, Tüzér Street 1, H-7623 Pécs, Hungary; ban.agnes@pte.hu; 3 Clinical Center, Department of Pathology, Medical School, University of Pécs, Szigeti Street 12, H-7624 Pécs, Hungary; kereskai.laszlo@pte.hu; 4 Janus Pannonius Clinical Block, Hematology Department, Clinical Center, University of Pécs, Ifjúság Road 13, H-7624 Pécs, Hungary; aszomor2@mail.fmkorhaz.hu; 5 Hematology Department, Szent György University Hospital, Seregélyesi Road 3, H-8000 Székesfehérvár, Hungary

**Keywords:** extranodal NK/T-cell lymphoma, nasal-type lymphoma, isolated oral manifestation, EBV-associated lymphoma, L-asparaginase therapy, multidisciplinary management

## Abstract

**Background/Objectives**: Extranodal NK/T-cell lymphoma, nasal type (ENKTCL-NT), is a rare and extremely aggressive subtype of non-Hodgkin lymphoma that most frequently involves the nasal cavity and upper aerodigestive tract. Primary isolated oral manifestation is exceptionally uncommon and may mimic odontogenic or infectious diseases, delaying diagnosis. We report a case of ENKTCL-NT presenting initially as a destructive oral lesion without sinonasal involvement at diagnosis. **Methods**: A 32-year-old man with progressive palatal ulceration underwent clinical and imaging assessment (panoramic radiography and staging ^18F-FDG PET–CT) and repeated biopsies. Diagnosis was established using histopathology (H&E), immunohistochemistry (T-cell markers and cytotoxic profile), EBV detection by EBER in situ hybridization, and T-cell receptor gamma (TCRG) gene rearrangement analysis. **Results**: The lesion presented as a hemorrhagic, ulcerative palatal destruction covered by pseudomembranous exudate and was complicated by fungal infection, periostitis, and severe dental inflammatory foci, contributing to diagnostic delay. Histopathological examination revealed extensive necrosis with a dense atypical lymphoid infiltrate; angiocentric and angiodestructive growth was identified in one biopsy specimen. Tumor cells expressed T-cell markers (CD2, CD3, CD5, CD7; heterogeneous) and cytotoxic markers (TIA-1) and showed CD30 and CD56 positivity, with EBV positivity confirmed by EBER in situ hybridization. Molecular analysis demonstrated monoclonal TCRG rearrangement, and Ki-67 indicated high proliferative activity. Initial PET–CT demonstrated an intensely FDG-avid, locally invasive lesion without distant organ involvement. The patient was treated with L-asparaginase-based SMILE chemotherapy followed by radiotherapy (50 Gy), achieving marked initial clinical improvement and partial metabolic response; however, systemic relapse subsequently occurred with refractory disease despite salvage therapy and immunotherapy. **Conclusions**: This case highlights the substantial diagnostic challenge posed by isolated oral extranodal NK/T-cell lymphoma, nasal type, which may closely mimic benign inflammatory or infectious conditions and lead to significant diagnostic delay. Persistent, progressive, or therapy-resistant oral ulcerations should prompt early consideration of hematologic malignancy. Timely biopsy with comprehensive immunophenotyping, EBV testing, and close multidisciplinary collaboration are essential for accurate diagnosis and may contribute to earlier diagnosis and improved patient outcomes in these rare and atypical presentations.

## 1. Introduction and Clinical Significance

Extranodal NK/T-cell lymphoma, nasal type (ENKTCL-NT), is a rare, extremely aggressive subtype of non-Hodgkin lymphoma, characterized by angiocentric and angiodestructive growth, extensive necrosis, and a consistent association with Epstein–Barr virus (EBV) infection [1,2,3].

Although the majority of cases arise in the nasal cavity or upper aerodigestive tract, the 5th edition of the WHO classification acknowledges that ENKTCL may originate at a wide variety of extranodal sites, including the skin, gastrointestinal tract, testis, and—very rarely—the oral cavity [4,5].

Primary mucosal involvement in the mouth represents a small minority of cases and is typically accompanied by sinonasal disease; truly isolated presentations are exceptionally uncommon [6,7,8].

The epidemiology of ENKTCL-NT demonstrates striking geographic variability. Incidence is highest in East Asia and Central/South America, likely influenced by genetic susceptibility, environmental exposures, chronic inflammation, and persistent EBV infection [9,10,11,12,13,14,15]. Occupational or chemical exposures have also been suggested as potential cofactors in disease development [14,15].

Because early ENKTCL-NT lesions frequently exhibit extensive necrosis and superimposed infection, they often clinically resemble severe inflammatory or odontogenic conditions, resulting in diagnostic delay. Necrosis may obscure viable tumor tissue, meaning multiple or deep biopsies are often required for definitive diagnosis [16,17].

### 1.1. Relevance to Dental and Oral–Maxillofacial Practice

Oral presentations of ENKTCL-NT may mimic a broad spectrum of dental and periodontal diseases, including necrotizing ulcerative gingivitis, fungal infections, periostitis/osteitis, traumatic injury, or progressive palatal ulceration [18,19]. Dentists, periodontists, and oral and maxillofacial surgeons are frequently the first clinicians to encounter these lesions. Typical red-flag features include:

- Persistent or rapidly progressing mucosal ulceration;
- Unexplained palatal or gingival destruction;
- Tooth mobility not explained by periodontitis;
- Soft tissue swelling not responding to conventional therapy.

Because these early manifestations are nonspecific, delays in biopsy and referral are common and may negatively impact prognosis [20,21]. Given that early diagnosis significantly improves survival outcomes, heightened awareness among dental professionals is essential.

### 1.2. Purpose of This Case Report

This report presents an exceptionally rare case of ENKTCL-NT manifesting exclusively as a destructive oral lesion without sinonasal involvement. The diagnosis was substantially complicated by overlapping fungal infection, periodontal disease, and recent dental trauma—factors that contributed to initial misinterpretation. By providing a detailed description of the clinical features, diagnostic pathway, histopathologic findings, and multidisciplinary decision-making, this case aims to support clinicians in recognizing ENKTCL-NT in its atypical oral forms and emphasizes the critical role of dental specialists in the early detection of hematologic malignancies.

### 1.3. Clinical Significance

This case underscores the importance of considering hematologic malignancy, specifically ENKTCL-NT, in patients presenting with persistent or non-healing ulcerative oral lesions unresponsive to antimicrobial or surgical therapy. Isolated oral involvement without sinonasal disease is exceptionally uncommon, which may obscure the underlying diagnosis and delay appropriate oncologic treatment. Early biopsy, with immunophenotyping and EBV detection, and close interdisciplinary collaboration are essential for timely identification and improved patient outcomes.

## 2. Case Presentation

### 2.1. Patient Information

A 32-year-old male patient was referred to our clinic with atypical dental complaints. He worked as a locksmith with continuous exposure to physical strain and chemical agents. His medical history included chronic smoking for approximately 15 years (22.5 pack-years). His father had died of lung cancer. Several weeks before presentation, the patient experienced a fall with transient loss of consciousness, during which he believed he might have injured his upper anterior teeth. Emergency evaluation attributed the episode to transient hypovolemia.

### 2.2. Clinical Findings

**Figure 1:**
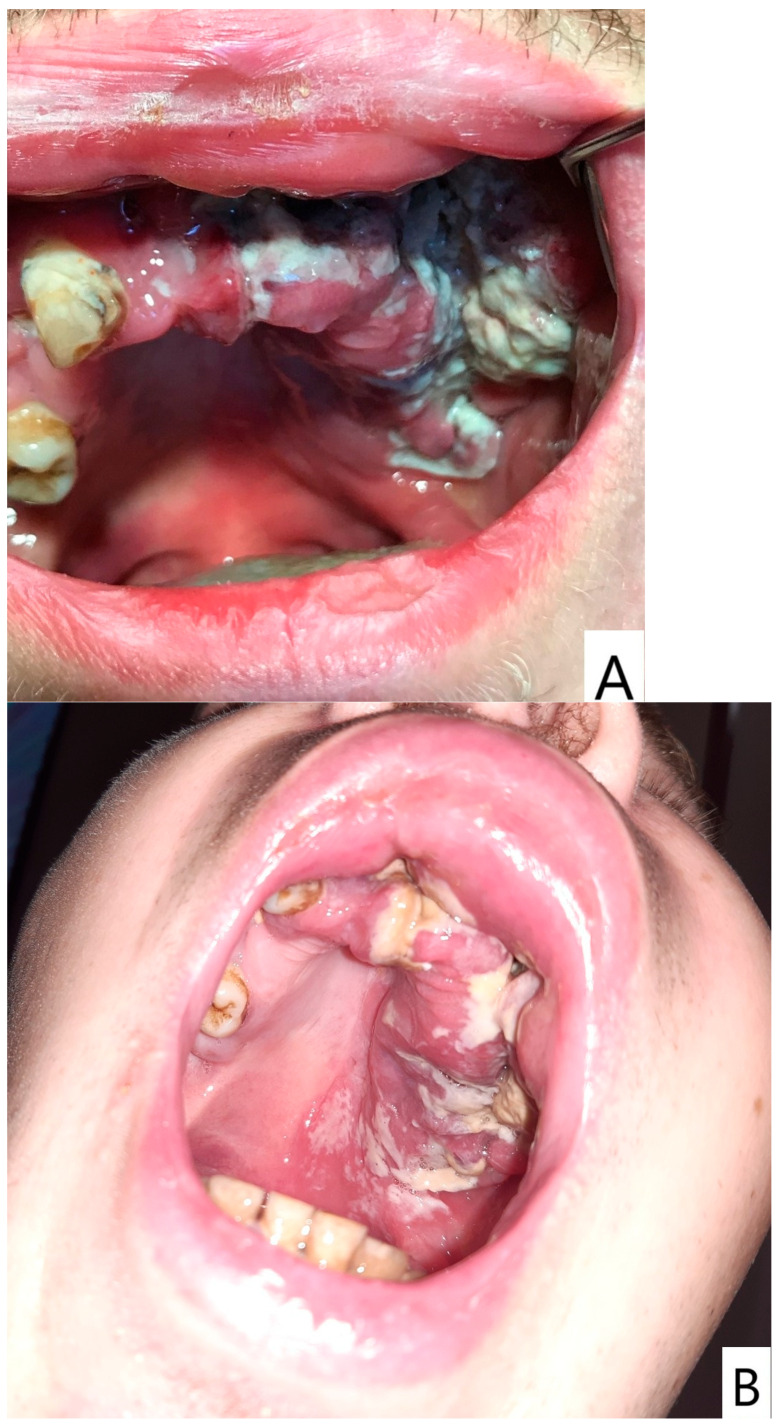
(**A**) Overview of the oral cavity demonstrating severe dental neglect and inflammatory changes. (**B**) Close-up view of the left palatal region showing a destructive ulcerative lesion with irregular margins, pseudomembranous covering, and hemorrhagic surface, representing the primary manifestation of extranodal NK/T-cell lymphoma in this case. Please note that several severely compromised maxillary teeth visible on the initial panoramic radiograph were extracted during acute dental management prior to photographic documentation; therefore, the intraoral images reflect the clinical status after initial emergency interventions.

**Figure 2:**
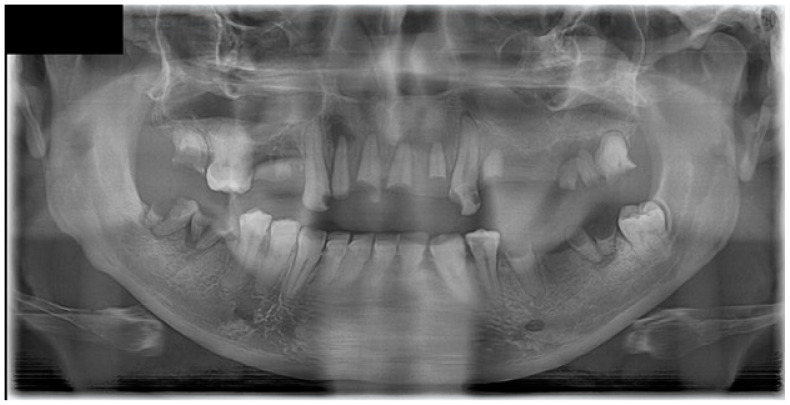
Panoramic radiograph (dental orthopantomogram, DOP) demonstrating extensive dental pathology and maxillary involvement. The image shows multiple residual roots, advanced carious destruction, generalized periodontal bone loss, and irregular osteolytic changes in the left maxillary alveolar process, corresponding to the clinically affected region. These findings initially supported an odontogenic and inflammatory etiology but did not fully explain the progressive, destructive soft tissue lesion.

Following a minor facial trauma, the patient developed nonspecific symptoms, including left midfacial discomfort, subjective nasal obstruction in the absence of sinonasal involvement, and dull facial pain. At the time of his first presentation to our clinic, the oral mucosa already showed advanced pathological changes. Physical and radiological examinations did not reveal traumatic injuries or findings that could adequately explain the severity of the clinical presentation (Figure 1 and Figure 2).

Intraoral examination at initial evaluation revealed an ulcerative, bleeding palatal lesion covered by pseudomembranous exudate, accompanied by surrounding inflammation. Additional findings included multiple root remnants, extensive carious lesions, periostitis, and severe dental neglect (Figure 1). Despite targeted ambulatory antibiotic therapy, antifungal treatment (fluconazole 100 mg twice daily), and local dental interventions, the lesion failed to regress and demonstrated further progression, with increasing tissue destruction involving the palate, left maxillary process, and adjacent gingival tissues. Periodontal swelling and pathological mobility of the maxillary teeth were also observed.

At initial presentation, the patient denied fever, dysphagia, night sweats, or weight loss. However, within one month, he developed unintentional weight loss of approximately 4 kg and new-onset night sweats. The persistence and progression of the destructive oral lesion despite antimicrobial and surgical management raised suspicion of an underlying malignant process. Initial clinical differential diagnosis included odontogenic infection with associated periostitis, invasive fungal infection, necrotizing periodontal disease, traumatic ulceration, granulomatous disease, and malignant processes, including hematologic malignancies such as lymphoma.

The progressive, therapy-resistant course of the lesion, extensive necrosis, and lack of sustained response to antimicrobial and antifungal treatment prompted repeated biopsies and further histopathological evaluation.

### 2.3. Diagnostic Assessment

Ten months after initial presentation revealed a similar mixed histological pattern, supporting a chronic, progressive disease course rather than an acute reactive process. Angiocentric and angiodestructive growth was identified in one of the biopsy specimens, consistent with the characteristic histomorphological features of extranodal NK/T-cell lymphoma.

A subsequent incisional biopsy obtained from the left maxillary alveolar and palatal region revealed extensive mucosal necrosis with an underlying dense, atypical lymphoid infiltrate. On hematoxylin and eosin (H&E) staining, the infiltrate consisted predominantly of medium-sized atypical lymphoid cells with hyperchromatic, irregular nuclei, coarse chromatin, scant cytoplasm, and frequent mitotic figures. The background showed prominent necrosis, acute inflammatory cells, and bacterial colonization.

**Figure 3:**
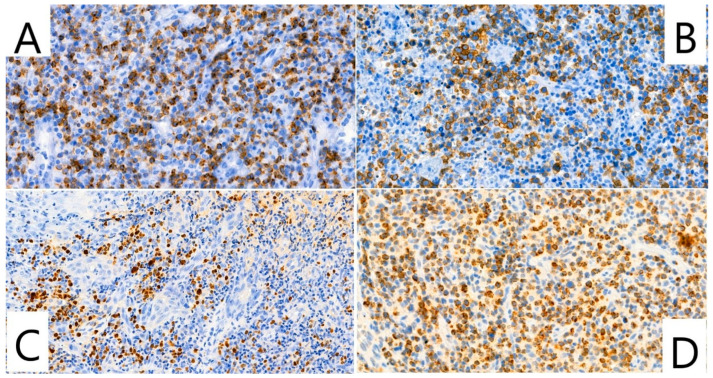
Extranodal NK/T-cell lymphoma, nasal type, demonstrating immunophenotypic features of the lesion. Immunohistochemical and in situ hybridization analyses: (**A**) CD2 positivity (IHC; original magnification ×40). (**B**) Strong CD30 expression (IHC; original magnification ×35). (**C**) Epstein–Barr virus positivity detected by EBER in situ hybridization (ISH; original magnification ×27). (**D**) Cytoplasmic expression of the cytotoxic marker TIA-1 (IHC; original magnification ×40). *Abbreviations:* IHC = immunohistochemistry; ISH = in situ hybridization.

In one biopsy specimen, the atypical lymphoid cells showed angiocentric growth with partial vessel wall destruction, consistent with angiodestructive behavior. Immunohistochemical analysis demonstrated expression of T-cell markers (CD2, CD3, CD5, CD7) with heterogeneous intensity (Figure 3A), most consistently for CD3. Strong expression of the cytotoxic markers TIA-1 and CD30 was observed (Figure 3B,D). CD56 was positive in the majority of tumor cells. B-cell markers CD20 and PAX5 were negative in the neoplastic population.

Epstein–Barr virus association was confirmed by EBER in situ hybridization, which demonstrated nuclear positivity in the atypical lymphoid cells (Figure 3C). Molecular analysis demonstrated a monoclonal T-cell receptor gamma (TCRG) gene rearrangement, supporting a malignant T-cell lineage. The proliferative activity was high, as assessed by Ki-67 immunostaining, indicating aggressive biological behavior.

At the request of the hematology team, additional immunohistochemical studies were performed for therapeutic stratification. PD-1 expression was detected only in a minimal fraction of cells, whereas PD-L1 immunostaining showed approximately 40% positivity within the overall cell population, including tumor cells, reactive lymphocytes, and macrophages. Due to the difficulty in unequivocally distinguishing tumor cells from reactive elements, a tumor proportion score (TPS) could not be reliably determined; however, the combined positive score (CPS) was calculated as 40%.

**Figure 4:**
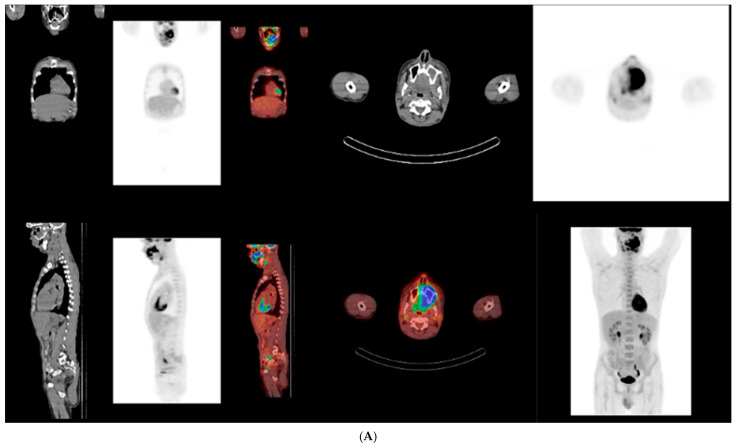

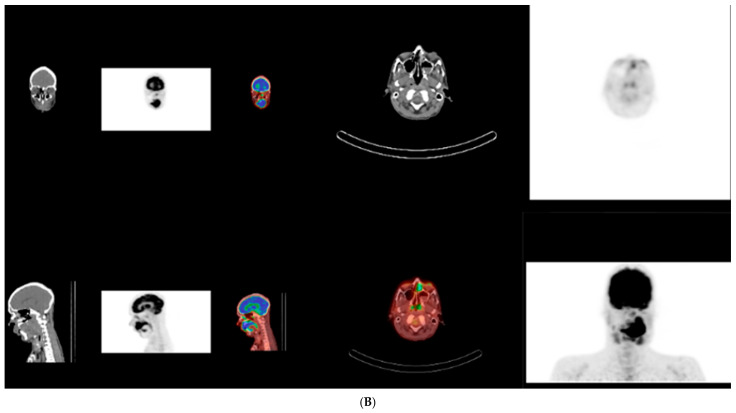
(**A**,**B**) Whole-body 18F-FDG PET–CT at initial staging showing an intensely FDG-avid, locally invasive lesion centered in the left maxillary and palatal region, extending to adjacent oral and maxillofacial soft tissues. The findings indicate aggressive local disease without evidence of distant organ involvement, pathological lymphadenopathy, bone marrow infiltration, or central nervous system involvement at the time of diagnosis. Regions demonstrating increased FDG uptake (warmer colors) reflect metabolically active disease.

Upon referral to hematology, PET–CT imaging demonstrated a metabolically active, locally invasive lesion confined to the oral and maxillofacial region, without evidence of distant organ involvement at initial staging (Figure 4). At the time of initial presentation, imaging assessment consisted of panoramic radiography and PET-CT, which was selected as the primary modality for staging and evaluation of disease extent in this aggressive lymphoma. No dedicated thin-slice CT was performed at initial diagnosis, as PET-CT provided comprehensive staging information. Bone marrow biopsy and cerebrospinal fluid analysis showed no evidence of lymphoma infiltration.

### 2.4. Therapeutic Intervention

The patient received combination chemotherapy according to the SMILE protocol (methotrexate, ifosfamide, etoposide, dexamethasone, L-asparaginase), along with intrathecal prophylactic triple therapy. A total of four chemotherapy cycles were administered, and 3D-conformal radiotherapy to a total dose of 50 Gy was performed after the second cycle.

Conservative dental therapy was prioritized whenever feasible. Periodontal treatment, focal dental sanitation, and correction of the sharp alveolar ridge using a piezoelectric spherical tip were performed under antibiotic coverage during multimodal oncologic treatment.

### 2.5. Follow-Up and Outcomes

**Figure 5:**
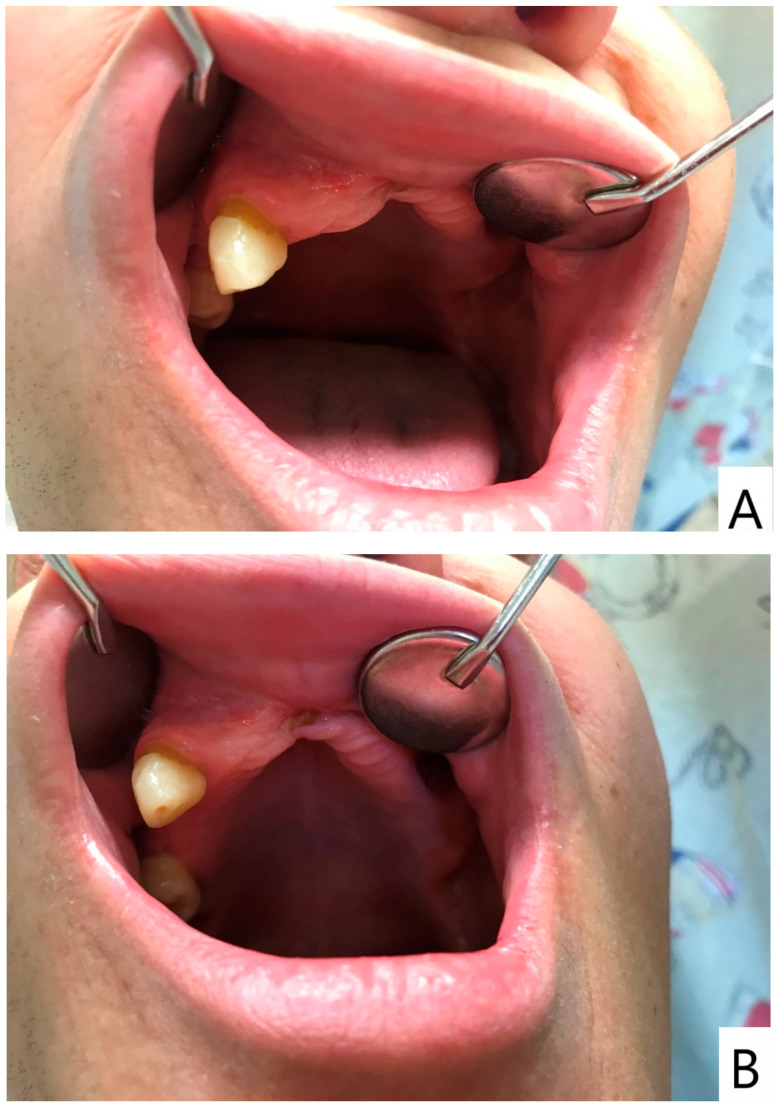
Oral manifestation of ENKTCL following clinical remission. (**A**) Intraoral view demonstrating marked regression of the previously ulcerative palatal lesion after completion of combined oncologic and supportive dental management. (**B**) Additional intraoral photograph obtained at the same follow-up visit from a different angle, confirming mucosal healing and absence of active ulceration.

**Figure 6:**
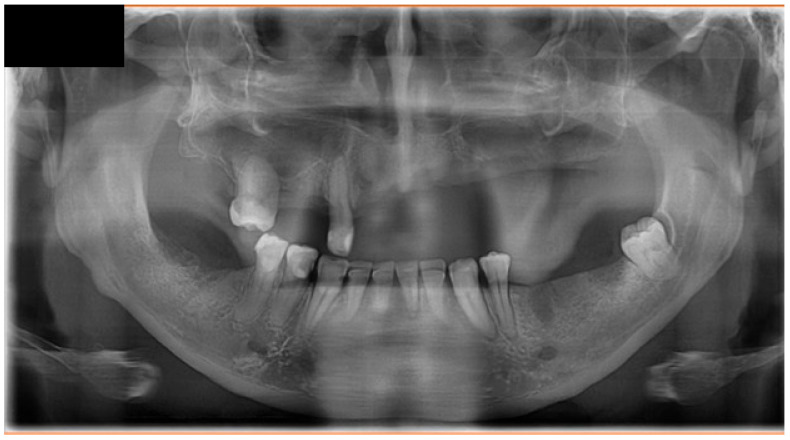
Digital orthopantomogram (DOP) obtained during clinical remission, demonstrating stabilization of maxillary structures following combined oncologic treatment and supportive dental management.

**Figure 7:**
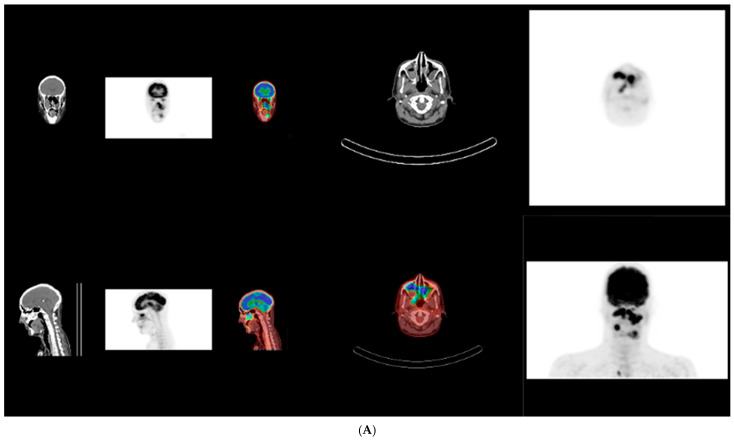

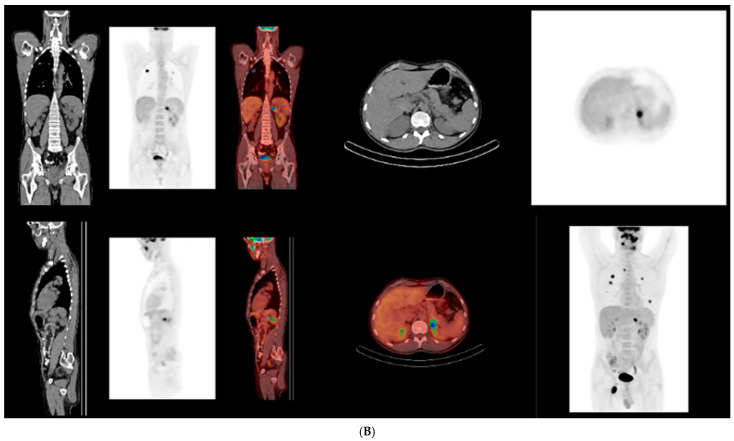
(**A**,**B**) ^18F-FDG PET–CT images obtained at the time of systemic relapse, demonstrating disseminated metabolically active disease involving nodal and extranodal sites.

Combined multimodal therapy resulted in a marked initial clinical improvement. During SMILE chemotherapy (weeks 6–10 from initial presentation), significant regression of the oral lesion was observed, accompanied by symptomatic relief (Figure 5 and Figure 6). Following completion of 3D-conformal radiotherapy to a total dose of 50 Gy (month 4), PET-CT demonstrated a partial metabolic and morphological response, consistent with temporary disease control. After a follow-up period of approximately three months (months 5–7) following completion of radiotherapy, the patient remained clinically stable, with no evidence of local progression. However, at approximately nine months from initial presentation, PET-CT revealed extensive systemic relapse, involving nodal, pulmonary, adrenal, cutaneous, and subcutaneous sites (Figure 7).

A peripherally inserted central catheter (PICC) was placed, and salvage therapy with DHAP (dexamethasone, cisplatin, cytarabine) combined with brentuximab vedotin was initiated. Despite treatment escalation and confirmed full sibling HLA compatibility, the patient’s clinical condition progressively deteriorated due to refractory disease and recurrent febrile neutropenia. Compassionate-use immunotherapy with nivolumab combined with oral venetoclax was subsequently administered; however, the disease remained entirely refractory.

The patient died approximately 11 months after initial presentation due to progressive disseminated disease. The chronological clinical course is summarized in Supplementary Table S1.

## 3. Discussion

Extranodal NK/T-cell lymphoma, nasal type (ENKTCL-NT), is an uncommon but clinically aggressive malignancy with a strong association to Epstein–Barr virus (EBV). Although ENKTCL-NT most frequently originates in the nasal cavity or upper aerodigestive tract, occurrences in the oral cavity are extremely rare, particularly as isolated lesions without sinonasal involvement. Recent multi-institutional studies of mature T/NK-cell neoplasms in the oral and maxillofacial region confirm that the palate is the most frequently affected oral site, but even in these cohorts, isolated oral cases remain exceptional and are usually accompanied by broader midfacial disease [8].

The diagnostic difficulty observed in our patient is consistent with prior reports describing deep necrotic ulcers, painful palatal destruction, and a broad differential diagnosis including fungal infections, granulomatous disease, traumatic lesions, and other lymphomas. Case reports of palate- or gingiva-origin ENKTCL similarly emphasize that early manifestations may mimic odontogenic infections or periodontal pathology, delaying biopsy and subsequent diagnosis [6,7]. In the present case, concurrent fungal infection, severe dental neglect, and periostitis contributed substantially to the initial misinterpretation and reflect the well-documented overlap between necrosis-driven inflammatory changes and true neoplastic infiltration.

Histopathologic confirmation remains the cornerstone of diagnosis. Necrosis frequently limits the availability of viable tumor tissue, and repeated or deep biopsies are often required. As in our case, the detection of cytotoxic markers (CD56, CD30, TIA-1) and EBV-encoded small RNAs via EBER in situ hybridization is essential for establishing ENKTCL-NT. Cytological clues such as azurophilic granules in atypical nonepithelial tumor cells, described by Niwa et al., may further assist in early differentiation from infectious or inflammatory ulcers.

Epstein–Barr virus (EBV) plays a central role in the pathogenesis of extranodal NK/T-cell lymphoma, nasal type. EBV-driven oncogenesis is mediated through latent viral gene expression, which interferes with host cell-cycle regulation, promotes G1/S phase transition, inhibits apoptosis, and facilitates immune evasion, processes that have been implicated in malignant transformation in EBV-associated lymphomas [5,22].

These mechanisms contribute to uncontrolled lymphoid proliferation and the aggressive biological behavior of the disease. Accordingly, detection of EBV by EBER in situ hybridization represents a critical diagnostic criterion and helps distinguish ENKTCL-NT from reactive or inflammatory EBV-associated lymphoproliferative disorders.

In addition to its diagnostic value, EBV positivity has important prognostic and therapeutic implications, as EBV-driven tumors are associated with aggressive clinical course and may show differential response to L-asparaginase-based chemotherapy and emerging immunotherapeutic approaches, as suggested by recent clinical and translational studies. Recent molecular studies have further elucidated the role of EBV latent genes in driving cell-cycle dysregulation and malignant transformation in EBV-associated lymphomas. Management of ENKTCL-NT typically requires combined chemoradiotherapy. Historical CHOP-based regimens, as described by Nikitakis et al., yield suboptimal outcomes due to the tumor’s intrinsic multidrug resistance. In contrast, L-asparaginase-based therapies, such as SMILE, now represent the preferred first-line approach for advanced disease and were applied in our patient. Despite an initial clinical response, the patient rapidly developed disseminated relapse, which is consistent with international data demonstrating poor outcomes in relapsed/refractory ENKTCL-NT and the limited efficacy of available salvage regimens, even when targeted agents or immunotherapy are applied.

This case reinforces the need for heightened clinical suspicion among dental and maxillofacial specialists. Persistent, destructive, or atypical oral ulcers—particularly those unresponsive to standard antimicrobial treatment—should prompt early biopsy, EBV testing, and multidisciplinary collaboration. The increasing number of published oral ENKTCL-NT cases highlights that early recognition in dental settings may improve time to diagnosis and treatment initiation, ultimately impacting survival.

From a differential diagnostic perspective, acute EBV-positive cytotoxic T-cell lymphoid hyperplasia of the upper aerodigestive tract and Epstein–Barr virus mucocutaneous ulcer (EBV-MCU) were considered. Acute EBV-positive cytotoxic T-cell lymphoid hyperplasia is generally regarded as a reactive and self-limited condition, characterized by preserved tissue architecture, lack of destructive angiocentric growth, absence of marked cytological atypia, and a polyclonal T-cell receptor gene rearrangement. In contrast, the present case demonstrated extensive tissue necrosis, angiocentric and angiodestructive growth, marked cytological atypia, and a monoclonal TCRG gene rearrangement, supporting a malignant lymphoid process.

EBV-MCU was also excluded, as this entity typically arises in the context of age-related or iatrogenic immunosuppression and presents as a sharply circumscribed, superficial ulcer with an indolent clinical course. Our patient was immunocompetent, and the lesion exhibited progressive, locally destructive behavior with deep tissue infiltration and aggressive clinical progression, which is not consistent with EBV-MCU. Taken together, the clinical course, histomorphological features, immunophenotype, EBV association, and molecular findings strongly support the diagnosis of extranodal NK/T-cell lymphoma, nasal type [4,5,12,22,23].

Beyond its diagnostic significance, the tumor immune microenvironment also plays an important role in disease progression and treatment response in extranodal NK/T-cell lymphoma. Immune checkpoint pathways, particularly the PD-1/PD-L1 axis, have been implicated in immune escape mechanisms and therapeutic resistance. Recent studies suggest that high PD-L1 expression, often in combination with other immune-regulatory molecules such as VISTA, is associated with adverse clinicopathological features, poor progression-free and overall survival, and limited response to PD-1 blockade in ENKTCL. These findings may be clinically relevant in the context of our case, as PD-1/PD-L1 immunohistochemical evaluation was performed for therapeutic decision-making, and the disease remained refractory despite checkpoint inhibitor therapy [23,24].

## 4. Conclusions

Isolated oral presentation of extranodal NK/T-cell lymphoma, nasal type (ENKTCL-NT), remains exceptionally rare and often mimics benign inflammatory or infectious conditions, which may significantly delay diagnosis. This case illustrates how overlapping factors-such as superimposed fungal infection, periodontal disease, and trauma-can obscure recognition of the underlying malignancy.

Persistent, progressive, or atypical oral ulcerations that fail to respond to conventional dental or antimicrobial therapy should prompt early biopsy, including immunophenotyping and EBV testing. Because the oral cavity may occasionally serve as the first and only site of ENKTCL-NT manifestation, dental professionals play a crucial role in early detection.

Timely pathological evaluation and close multidisciplinary collaboration between dentistry, pathology, hematology, and radiology are essential for accurate diagnosis and optimal patient outcomes. Continued awareness of this rare but aggressive entity may contribute to earlier recognition and improved management of patients presenting with unusual oral lesions.

## Data Availability

The original contributions presented in this study are included in the article and Supplementary Materials. Further inquiries can be directed to the corresponding author.

## Supplementary Materials

The following supporting information can be downloaded at: https://www.mdpi.com/article/10.3390/dj14020129/s1, Table S1: Chronological timeline of clinical course, diagnostic procedures, treatments, and outcomes.

## Abbreviations

The following abbreviations are used in this manuscript:

CAM	Cell adhesion molecule
COO	Cell of origin
DOP	Digital Orthopantomogram
EBER	EBV-encoded small RNA
EBV	Epstein–Barr virus
EBV-MCU	Epstein–Barr virus muco-cutaneous ulcer
ENKTCL-NT	Extranodal NK-/T-cell lymphoma, nasal type
NHL	Non-Hodgkin lymphoma
NK-cell	Natural killer cell
PD-1	Programmed cell death protein-1
PD-L1	Programmed death-ligand 1
PICC	Peripherally inserted central catheter
VISTA	V-domain Ig suppressor of T-cell activation
WHO	World Health Organization
