# Supplementary material for: Extranodal NK/T-Cell Lymphoma, Nasal Type, Presenting as an Isolated Oral Manifestation
Source: Dent J (Basel). 2026 Feb 23;14(2):129. doi: 10.3390/dj14020129 (PMC12939484; doi:10.3390/dj14020129)
Supplement: Supplementary file 1 [file dentistry-14-00129-s001.zip › dentistry-4049020-supplementary.pdf]

Table S1. Timeline of clinical course, diagnostic work-up, and treatment

Time from initial presentation	Clinical events
**Week 0**	Initial presentation with facial swelling and palatal ulceration following reported trauma.
**Weeks 1–2**	Hospital admission; dental extractions; initial biopsies showing extensive necrosis and suspected fungal infection.
**Weeks 3–4**	Persistent progression despite antimicrobial therapy; repeat incisional biopsy performed.
**Week 5**	Histopathological, immunohistochemical, and molecular studies confirmed extranodal NK/T-cell lymphoma, nasal type.
**Weeks 6–10**	Initiation of SMILE chemotherapy with marked clinical regression of the oral lesion.
**Month 4**	Completion of radiotherapy (total dose 50 Gy) to the maxillofacial region.
**Months 5–7**	Temporary clinical remission; partial metabolic response on PET-CT.
**Month 9**	Systemic relapse with disseminated disease documented on PET-CT imaging.
**Months 9–11**	Salvage therapies initiated; disease remained refractory.
**Month 11**	Patient deceased due to progressive disseminated disease.
